# The Relationship Between 2D:4D Digit Ratio and Polycystic Ovary Syndrome: A Systematic Review

**DOI:** 10.1002/ajhb.70288

**Published:** 2026-06-06

**Authors:** Tuba Güner Emül, Emine Kaplan Serin

**Affiliations:** ^1^ Department of Nursing, Obstetrics, Women's Health and Gynaecological Diseases Mersin University Mersin Turkey; ^2^ Faculty of Nursing, Department of Internal Medicine Nursing Mersin University Mersin Türkiye

**Keywords:** 2D:4D ratio, digit ratio, polycystic ovary syndrome, prenatal androgens

## Abstract

**Objectives:**

This systematic review aimed to synthesize current evidence on the association between 2D:4D ratios and PCOS.

**Methods:**

A comprehensive literature search was performed in PubMed, Web of Science, Scopus, and Cochrane Library databases between May and June 2025. Two independent researchers conducted study screening, data extraction, and quality appraisal using the Joanna Briggs Institute critical appraisal tools.

**Results:**

Eleven studies published between 2005 and 2024, with sample sizes ranging from 40 to 400 participants, were included. Study designs were cross‐sectional or case–control, and measurement techniques involved calipers, photocopies, or digital analysis. Nine studies reported significantly lower 2D:4D ratios in women with PCOS compared to healthy controls, supporting the hypothesis of increased prenatal androgen exposure. However, two studies found no significant association, likely due to methodological heterogeneity, small sample sizes, or diagnostic inconsistencies.

**Conclusion:**

The majority of included studies suggest that lower 2D:4D ratios may reflect elevated prenatal androgen exposure in women with PCOS, indicating its potential as a non‐invasive biomarker. Nonetheless, variability in diagnostic criteria, measurement methods, and study populations limits generalizability. Further large‐scale, standardized research is required to determine clinical utility and to establish population‐specific reference values.

## 1 Introduction

Polycystic Ovary Syndrome (PCOS) is an endocrine disorder affecting women of reproductive age, characterized by chronic anovulation, hyperandrogenism, reduced fertility, and menstrual irregularities (Can and Türker 2023; Zeng et al. 2020). Although its exact etiology remains unclear, PCOS affects approximately 6%–10% of women of reproductive age (Zeng et al. 2020). Hormonal profiles of individuals with PCOS typically show elevated levels of luteinizing hormone (LH), prolactin, and androgens. Additionally, insulin resistance and its associated complications, such as type 2 diabetes mellitus, hypertension, and metabolic syndrome, represent significant health concerns in this population (Zeng et al. 2020; Witchel Selma, Burghard, et al. 2019; Witchel Selma, Sharon, et al. 2019). The primary problem in PCOS pathogenesis is compensatory hyperinsulinemia arising from insulin resistance. Other contributing factors include genetic predisposition, beta‐cell dysfunction, abnormal ovarian and adrenal steroidogenesis, alterations in the steroid metabolome, neuroendocrine and environmental influences, epigenetic modifications, and impaired responses to energy surplus or restriction (Teede et al. 2023; Witchel Selma, Burghard, et al. 2019; Witchel Selma, Sharon, et al. 2019). Recent evidence highlights the role of functional ovarian hyperandrogenism as a central mechanism, while genetic, metabolic, and environmental interactions also contribute (Teede et al. 2023). Importantly, prenatal androgen exposure has been implicated in the developmental origins of PCOS, with recent findings demonstrating transgenerational susceptibility mediated by in utero androgen exposure (Teede et al. 2023; Witchel Selma, Burghard, et al. 2019; Witchel Selma, Sharon, et al. 2019). The most recent 2023 International Evidence‐Based Guideline also underscores the multifactorial nature of PCOS pathogenesis, integrating metabolic, endocrine, genetic, and environmental determinants (Karakoç 2024). High androgen exposure in female fetuses is known to play a role in the later development of PCOS. This exposure may affect steroid production, insulin signaling, hypothalamic–pituitary interactions, neuroendocrine secretion patterns, and epigenetic factor modifications.

The digit ratio, specifically the ratio of the index finger to the ring finger (2D:4D), serves as an anatomical marker used to estimate prenatal androgen exposure. The 2D:4D ratio has been associated with various traits and conditions, including autism, dementia, personality traits, abilities, sexual orientation, and multiple diseases (Hönekopp and Watson 2010; Holden and Fekken 2020; Manning and Fink 2023). Higher digit ratios in individuals are generally linked to traits more commonly observed in females, supporting the view that the 2D:4D ratio is an indicator of prenatal testosterone exposure (Hönekopp and Watson 2010). The 2D:4D ratio is considered an indicator of first‐trimester prenatal hormonal exposure; specifically, higher estrogen and lower testosterone exposure are associated with a higher 2D:4D ratio (Holden and Fekken 2020). Zheng and Cohn (2011) described the developmental basis of sexually dimorphic digit ratios, supporting the use of 2D:4D as a proxy for prenatal hormonal influence.

These findings highlight the importance of systematically reviewing the literature to clarify the relationship between the 2D:4D digit ratio and Polycystic Ovary Syndrome (PCOS). Although several studies have investigated the association between the 2D:4D digit ratio and PCOS, the findings remain inconsistent and sometimes contradictory. While many studies report significantly lower 2D:4D ratios in women with PCOS, suggesting increased prenatal androgen exposure, other studies have failed to identify such an association. In addition, differences in study populations, diagnostic criteria, digit measurement techniques, and sample characteristics have limited the comparability of findings across studies. Therefore, a systematic synthesis of the available evidence is necessary to evaluate the consistency of the reported associations and to determine whether the 2D:4D ratio may serve as a reliable anthropometric marker for PCOS risk. Accordingly, the aim of this systematic review is to comprehensively evaluate the current evidence regarding the association between PCOS and the 2D:4D digit ratio.

## 2 Materials and Methods

The protocol for the systematic review and the writing of the article followed the PRISMA‐P (Preferred Reporting Items for Systematic Reviews and Meta‐Analyses Protocols) guidelines (Haddaway et al. 2022). It was registered in PROSPERO (CRD420251111940). The literature search, selection of articles, and data extraction were independently reviewed by the authors (T.G.E., E.K.S.).

**FIGURE 1:**
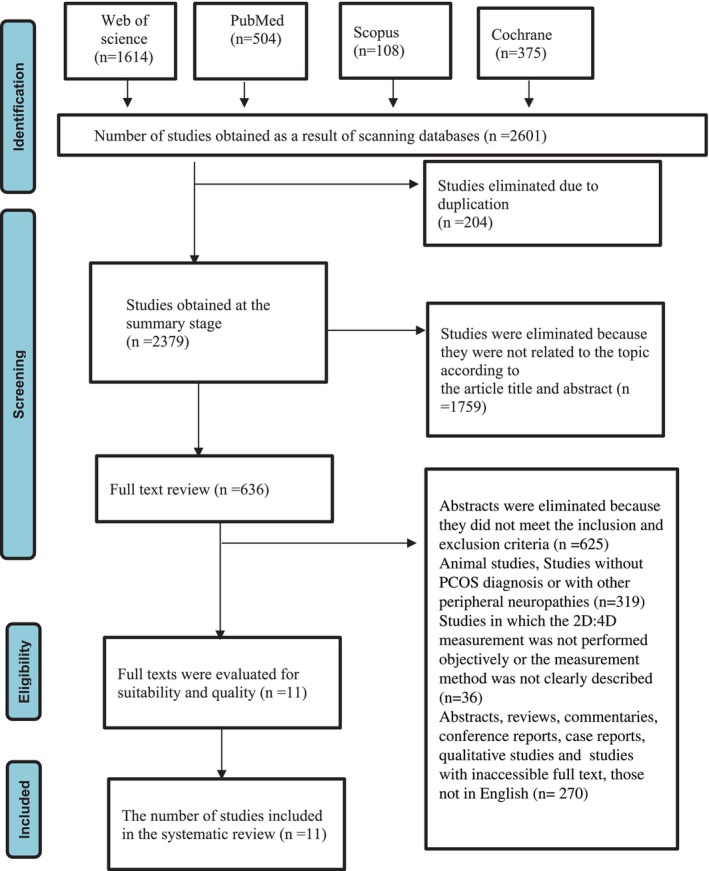
PRISMA flow chart of the screening process.

A comprehensive literature search was conducted for this systematic review. Databases including PubMed, Web of Science, Scopus, and Cochrane Library were used. The search was performed between May and June 2025. Keywords and MeSH terms such as “Polycystic Ovary Syndrome,” “PCOS,” “digit ratio,” “finger length,” and “2D:4D” were combined. An example of the search strategy is as follows:

The search was limited to studies published in English. Additionally, only studies conducted on humans were included, while animal studies and pediatric studies were excluded. All records obtained from the databases were independently screened by two researchers at the title and abstract level, and studies deemed appropriate were selected for full‐text review. The process of accessing the 11 studies included in the systematic review, out of a total of 2601 studies, is shown in the PRISMA flow Chart (Figure 1). Thanks to the advanced search engines of the databases, the process of identifying 2601 articles was carried out systematically and efficiently. The high number of records retrieved is an expected and common situation in systematic reviews, especially when a broad and inclusive search strategy is used. Broad terms were deliberately chosen to ensure that all potentially relevant studies were captured. Title and abstract screening became easier and more manageable due to the filtering and organization tools provided by the databases. Each stage of the screening process was carried out independently by two researchers to ensure accuracy and objectivity.

In this systematic review, the titles and abstracts of the articles were reviewed, and studies unrelated to the systematic review or duplicate articles were excluded. The selection of studies included in this systematic review was determined according to the inclusion criteria created by the PICOS method (Table 1) (Centre for Reviews and Dissemination 2008). When necessary, full‐text articles were reviewed according to the inclusion criteria. At the end of this process, 11 articles met the inclusion criteria and were included in the systematic review. The flow diagram explaining the selection of studies according to the PRISMA guidelines is presented (Figure 1). In the review, the titles and abstracts of the studies were initially read and assessed, and then the full‐text articles were examined in detail. The selections made independently by the researchers were compared, and a consensus was reached on differing opinions. To summarize the data, the authors developed a standard data extraction form, and the data were evaluated accordingly. The content of the data extraction form included information about the authors, year of publication, location of the study, study design, sample size, measurement tools used, and the results. In this systematic review, the methodological quality of the studies was assessed independently by the first and second researchers (TGE, EKS). The researchers' independent assessments were then compared and a consensus was reached in case of disagreement. In this review, we used the Joanna Briggs Institute (JBI) critical appraisal checklist, which is an internationally recognized tool widely applied in systematic reviews for assessing methodological quality and risk of bias, particularly for cross‐sectional studies. The methodological quality of the included studies was independently assessed by two reviewers using the Joanna Briggs Institute (JBI) Critical Appraisal Checklists appropriate for each study design. For cross‐sectional studies, the JBI Analytical Cross‐Sectional Studies Checklist (8 items) was used, whereas for case–control studies, the JBI Case–Control Checklist (10 items) was applied (Barker et al. 2023). Although JBI recommends descriptive reporting rather than the use of an overall score, for the purpose of summarizing methodological quality across the included cross‐sectional studies, we additionally calculated an 8‐point summary score, assigning 1 point for each “yes” response and 0 points for “no,”, “unclear,” or “not applicable” responses. The scores ranged from 6 to 8.

**TABLE 1:** Inclusion and exclusion criteria.

	Inclusion criteria	Exclusion criteria
P	Studies involving diagnosed with Polycystic Ovary Syndrome, confirmed clinically.	Animal studies. Studies without PCOS diagnosis.
İ	Studies assessing the 2D:4D digit ratio (the length ratio of the second and fourth digits), including both right and left hand measurements.	—
C	—	—
O	Studies that measured the 2D:4D digit ratio and explicitly reported these data.	Studies in which the 2D:4D measurement was not performed objectively or the measurement method was not clearly described.
S	Descriptive, cross‐sectional, prospective, retrospective, comparative, and randomized controlled English studies with accessible full text.	Abstracts, reviews, commentaries, conference reports, qualitative studies, case reports, and studies with inaccessible full text and those not in English.

## 3 Results

The systematic review a total of 11 studies published between 2005 and 2024 met the inclusion criteria, with sample sizes ranging from 40 to 400 participants. All included studies were case–control or cross‐sectional in design. The diagnosis of polycystic ovary syndrome (PCOS) was established using the Rotterdam criteria in most studies (Cattrall et al. 2005; Çatak et al. 2024; Deepika and Preethy 2021; Lujan, Podolski, et al. 2010; Lujan, Terri, et al. 2010; Roy et al. 2018; Yan et al. 2023; Yıldız et al. 2015), while a few employed the NIH criteria (Eltaweel et al. 2018; Pandit et al. 2016) or the Androgen Excess Society (AES) criteria (Evin and Ayrancı 2024). The measurement of the second‐to‐fourth digit ratio (2D:4D) varied across studies, including direct caliper measurements, photocopies, and digital/computer‐based analyses. Reports related to the studies are presented in Table 2.

**TABLE 2:** Studies reporting.

References (first author/year)	Desing of study/study groups	Assessment tool	Conclusion	Quality
Cattrall et al. (2005)/Australia.	A case control study. 70 women with PCOS aged 18–40 were included in the study.	This was performed using Vernier calipers measuring to 0.01 mm. Measurements were made directly with Vernier calipers rather than from hand photocopies.	Here we show a subtle difference in the finger length pattern of women with PCOS. This may constitute anatomical evidence of in utero androgen exposure in PCOS.	**7**
Lujan, Podolski, et al. (2010) and Lujan, Terri, et al. (2010)/USA.	This cross‐sectional study included. Digit ratios were determined in 98 women diagnosed with PCOS by the 2003 international consensus guidelines and in 51 women with regular menstrual cycles, no clinical or biochemical signs of hyperandrogenism and normal ovarian morphology.	Women with PCOS were divided into four clinical phenotypes (i.e., Frank, Non‐PCOS, Ovulatory, and Mild) and compared between groups with 2D:4D Tukey–Kramer multiple comparison tests.	Women with PCOS do not demonstrate finger length patterns that are consistent with increased prenatal androgen exposure. These findings do not preclude a role for prenatal androgens in the development of PCOS; however, low 2D:4D are not a characteristic of PCOS.	**7**
Lujan, Podolski, et al. (2010) and Lujan, Terri, et al. (2010)/USA.	Ninety six women diagnosed with PCOS according to the 2003 Rotterdam criteria had their finger lengths measured with computer‐assisted analysis. Participants were categorized into four recognized phenotypes of PCOS and their 2D:4D compared to healthy female controls (*n* = 48) and men (*n* = 50).	Measurements were made directly with Vernier calipers.	Computer‐assisted measurements validated that digit ratios of women with PCOS do not show anatomical evidence of increased prenatal androgen exposure.	**8**
Yıldız et al. (2015)/Türkiye.	This cross‐sectional study included 137 women. The mean age of our patients was 46.1 years.	The lengths of the second and fourth fingers, as well as blood pressure measurements, were taken.	The difference and ratio between the second and fourth finger lengths showed a weak correlation with total testosterone levels and a positive correlation with free testosterone levels. The easily measurable difference and ratio of the second and fourth finger lengths can be used as an important indicator of hyperandrogenism in women.	**7**
Pandit et al. (2016)/India.	400 females (200 cases and 200 controls) of age group 18–40 were studied.	The length of the index (2D) and ring finger (4D) were measured and 2D:4D ratio were calculated and statistically analyzed. Physical measurements were made from the ventral angle using Vernier Calipers.	This anatomical expression can be used as a tool for early prediction of PCOS and hence substantiates the need for lifestyle modification to counteract this syndrome at its nascent stage	**7**
Eltaweel et al. (2018)/Egypt.	The Cross‐sectional study 50 female. The current study was carried out on 50 female patients with hirsutism their ages ranged from 20 to 40 years with a mean age of 30.35 ± 6.02 years. The control group included 30 healthy, age‐matched women. Their ages ranged from 20 to 42 years with a mean age of 28.93 ± 6.20 years.	2D:4D ratios were measured and compared with metabolic syndrome–related parameters and body mass index (BMI).	In the study, it was determined during the history‐taking process that 24% of the patients with hirsutism had a history of polycystic ovary syndrome (PCOS). No statistically significant relationship was found between the 2D:4D ratio and PCOS for either the right hand (*p* = 0.544) or the left hand (*p* = 0.689).	**6**
Roy et al. (2018)/India.	The Cross‐sectional study 251 women of reproductive age group (15–45 years)	2D:4D ratios were measured.	After obtaining a statistically significant difference (*p* < 0.05) between 2D:4D ratio of cases and controls, a cut off value of 0.9928 for left hand with sensitivity 68.92 and specificity 72.98 and for right hand a cut off value of 0.9846 with sensitivity 66.53 and specificity 83.51 were determined, by interpreting 2D:4D of cases and controls using the ROC curve analysis.	**8**
Deepika and Preethy (2021)/India.	The study has an analytical cross‐sectional design. According to inclusion and exclusion criteria, a total of 48 participants were selected, with 24 individuals in the control group and 24 in the PCOS group, and their basic characteristics were recorded.	The lengths of the second and fourth fingers were directly measured, along with weight and height, and body mass index (BMI) was calculated.	This study demonstrates that the 2D:4D digit ratio decreases while BMI, body fat, and visceral fat significantly increase in the PCOS group.	**7**
Yan et al. (2023) China.	The study cross‐sectional study included 34 women without PCOS, 116 women with PCOS, and 40 men.	All finger length ratios of the right and left hands (2D:3D, 2D:4D, 2D:5D, 3D:4D, 3D:5D, and 4D:5D) were systematically measured.	In men, the left‐hand 2D:3D, 2D:4D, and 2D:5D ratios were significantly lower compared to women without PCOS. In women with PCOS, significant reductions in the left‐hand 2D:3D and 2D:4D ratios were observed compared to women without PCOS. In the subgroup analysis, the left‐hand 2D:3D and 2D:5D ratios of the hyperandrogenism subgroup were lower than those of the non‐hyperandrogenism subgroup, although these differences were not statistically significant. Logistic regression analysis indicated that the left‐hand digit ratios (2D:3D, 2D:4D, 2D:5D, and 3D:4D) were statistically associated with a diagnosis of PCOS.	**7**
Çatak et al. (2024)/Türkiye.	Cross‐sectional study. A total of 124 individuals (62 PCOS patients and 62 controls) participated in study. In PCOS group mean age was 24.30 ± 4.99 years, the control group mean age was 23.75 ± 2.83 years (*p* = 0.772).	The second and fourth finger length of all participants were measured in centimeters using a digital vernier caliper.	Finally, we examined 2D/4D finger ratios in PCOS patients. Prenatal testosterone activity affects 2D/4D lengths and their ratio in both hands.	**7**
Evin and Ayrancı (2024)/Türkiye.	Cross‐sectional study. The study included 38 adolescent girls with PCOS and 40 healthy adolescent girls as the control group. The mean age of the PCOS group was 15.99 ± 1.18 years, while that of the control group was 16.02 ± 1.06 years.	The 2D:4D ratio was calculated for both hands. Finger lengths were measured as the distance between the pseudophalangion and dactylion (da) points. Each measurement was taken twice on both hands using a digital caliper, and the average value was used.	The 2D:4D digit ratio in the right hand was found to be significantly lower in the PCOS group (0.93 ± 0.02) compared to the control group (1.00 ± 0.01) (*p* < 0.001). This study demonstrates that the 2D:4D ratios of both hands in patients with PCOS are significantly lower compared to healthy controls, suggesting prenatal androgen exposure.	**8**

In Australia, Cattrall et al. (2005) conducted a case–control study including 70 women with PCOS aged 18–40 and healthy controls. Direct Vernier caliper measurements were used, and lower right‐hand 2D:4D ratios were observed in women with PCOS, suggesting potential anatomical evidence of prenatal androgen exposure.

Lujan, Podolski, et al. (2010) and Lujan, Terri, et al. (2010) in the USA conducted a cross‐sectional study including 98 women diagnosed with PCOS according to the 2003 international consensus guidelines and 51 women with regular menstrual cycles as controls. Participants were divided into four clinical phenotypes, and 2D:4D ratios were compared. The findings showed no consistent finger length pattern indicative of increased prenatal androgen exposure in PCOS. Lujan, Podolski, et al. (2010) and Lujan, Terri, et al. (2010) measured finger lengths of 96 women with PCOS according to Rotterdam criteria using computer‐assisted analysis. Results were compared with healthy female (*n* = 48) and male (*n* = 50) controls. Direct caliper measurements confirmed that digit ratios in women with PCOS did not provide anatomical evidence of increased prenatal androgen exposure.

In Turkey, Yıldız et al. (2015) included 137 participants. The difference and ratio between the second and fourth finger lengths showed a weak correlation with total testosterone and a positive correlation with free testosterone (*p* < 0.05). A weak negative correlation was found with sex hormone‐binding globulin (SHBG) levels. No significant association was found with metabolic syndrome parameters. Pandit et al. (2016) in India studied 400 females (200 cases, 200 controls) aged 18–40. 2D:4D ratios were measured using Vernier calipers and suggested as a potential anatomical biomarker for early prediction of PCOS. Eltaweel et al. (2018) in Egypt conducted a cross‐sectional study with 50 women with hirsutism and 30 controls. No significant relationship was found between 2D:4D ratios and PCOS for either the right hand (*p* = 0.544) or left hand (*p* = 0.689). Roy et al. (2018) in India measured 2D:4D ratios in 251 women aged 15–45 and determined cutoff values using ROC analysis, indicating that lower ratios were associated with a higher probability of developing PCOS in adulthood. Deepika and Preethy (2021) conducted an analytical cross‐sectional study including 24 PCOS patients and 24 controls. BMI and body fat were significantly higher, and 2D:4D ratios were significantly lower in the PCOS group (*p* < 0.01). Yan et al. (2023) measured six different digit ratios in 34 healthy women, 116 women with PCOS, and 40 men. In women with PCOS, left‐hand 2D:3D and 2D:4D ratios were significantly lower. Logistic regression showed that left‐hand 2D:3D, 2D:4D, 2D:5D, and 3D:4D ratios were significantly associated with PCOS diagnosis. Çatak et al. (2024) in Turkey included 62 PCOS patients and 62 controls. Second and fourth finger lengths were measured using a digital caliper, and prenatal testosterone activity was suggested to influence 2D:4D ratios in both hands. Evin and Ayrancı (2024) included 38 adolescent girls with PCOS and 40 controls. 2D:4D ratios were significantly lower in both hands of the PCOS group. A moderate negative correlation was found between left‐hand 2D:4D and the modified Ferriman‐Gallwey score (*r* = −0.53, *p* = 0.01). Overall, nine out of the 11 studies reported significantly lower 2D:4D ratios in women with PCOS, supporting the potential role of prenatal androgen exposure in PCOS etiology. Heterogeneity in measurement methods and study populations limits the generalizability of these findings.

## 4 Discussion

This systematic review evaluated the current evidence regarding the association between the 2D:4D digit ratio and Polycystic Ovary Syndrome (PCOS). Overall, most included studies demonstrated significantly lower 2D:4D ratios in women with PCOS compared to healthy controls, supporting the hypothesis that increased prenatal androgen exposure may contribute to the developmental origins of PCOS.

The 2D:4D ratio has long been considered an indirect anatomical marker of prenatal hormonal exposure, particularly fetal testosterone levels. The predominance of lower digit ratios observed across the included studies aligns with current theories suggesting that prenatal androgen exposure may influence ovarian function, neuroendocrine regulation, and metabolic pathways associated with PCOS development. Studies conducted by Cattrall et al. (2005), Pandit et al. (2016), Roy et al. (2018), Deepika and Preethy (2021), Yan et al. (2023), Çatak et al. (2024), and Evin and Ayrancı (2024) generally reported lower 2D:4D ratios among women with PCOS, further strengthening the evidence suggesting a developmental role of prenatal androgen exposure in PCOS.

Although the majority of studies supported this association, Lujan, Podolski, et al. (2010), Lujan, Terri, et al. (2010), and Eltaweel et al. (2018) did not identify significant relationships between 2D:4D ratios and PCOS. These inconsistencies may be explained by methodological differences in digit measurement techniques, variability in PCOS diagnostic criteria, ethnic diversity, limited sample sizes, and heterogeneity among PCOS phenotypes. In addition, Eltaweel et al. (2018) included women with hirsutism rather than exclusively women with confirmed PCOS diagnoses, which may have influenced the findings. Similarly, Lujan, Podolski, et al. (2010) and Lujan, Terri, et al. (2010) emphasized that low 2D:4D ratios were not consistently observed across different PCOS phenotypes.

Several studies additionally examined anthropometric and metabolic parameters including body mass index (BMI), waist circumference, waist‐to‐hip ratio, blood pressure, and age. Yıldız et al. (2015) reported weak positive correlations between the 2D:4D ratio and total/free testosterone levels, as well as a weak negative correlation with sex hormone‐binding globulin (SHBG). Deepika and Preethy (2021) also found significantly higher BMI, body fat, and visceral fat values in women with PCOS alongside lower 2D:4D ratios. These findings suggest that lower digit ratios may be more strongly associated with prenatal hormonal exposure than with postnatal metabolic disturbances.

Pandit et al. (2016) and Roy et al. (2018) proposed that the 2D:4D ratio may serve as an early anthropometric marker for identifying individuals at increased risk of developing PCOS. Similarly, Yan et al. (2023) demonstrated that several left‐hand digit ratios were significantly associated with PCOS diagnosis and clinical hyperandrogenism. Evin and Ayrancı (2024) further reported a moderate negative correlation between left‐hand 2D:4D ratios and modified Ferriman–Gallwey scores in adolescents with PCOS, suggesting a relationship between digit ratio and clinical manifestations of hyperandrogenism.

Taken together, the findings indicate that the 2D:4D ratio may have potential as a non‐invasive and inexpensive anthropometric marker for identifying individuals at increased risk of PCOS. However, current evidence remains insufficient to support its use as a standalone diagnostic biomarker in clinical practice. Instead, the 2D:4D ratio may be more useful when evaluated together with clinical, hormonal, and metabolic indicators, particularly in adolescent populations where early identification and intervention may reduce long‐term reproductive and metabolic complications.

Importantly, substantial heterogeneity among studies limits the generalizability of current findings. Differences in measurement methods (direct caliper measurements, photocopies, digital analysis), ethnicity, age groups, and diagnostic approaches complicate comparisons across studies. Moreover, the indirect nature of the 2D:4D ratio as a marker of prenatal androgen exposure should be interpreted cautiously, since direct hormonal and genetic mechanisms remain insufficiently understood.

Healthcare professionals may contribute to early risk assessment in adolescents with suspected PCOS by considering anthropometric indicators together with clinical and metabolic findings. In addition, patient education and lifestyle interventions remain important components of PCOS prevention and management strategies. Future large‐scale, multi‐center, and cross‐national studies using standardized PCOS diagnostic criteria and uniform digit measurement protocols are needed. Establishing population‐specific reference values may further clarify the clinical utility of the 2D:4D ratio in PCOS research and screening.

## 5 Conclusion

The findings of this systematic review suggest that women with PCOS generally demonstrate lower 2D:4D digit ratios, supporting the hypothesis that prenatal androgen exposure may contribute to PCOS pathogenesis. Although the 2D:4D ratio appears promising as a non‐invasive anthropometric marker, current evidence remains insufficient for clinical application due to methodological heterogeneity and inconsistent findings across studies. Standardized, large‐scale, multi‐ethnic longitudinal studies are needed to clarify causality, improve comparability, and establish population‐specific reference values for potential future clinical use.

### 5.1 Limitations

There are several limitations that should be considered when interpreting the findings of this systematic review. First, the included studies exhibited heterogeneity in terms of study populations, sample sizes, measurement methods, and ethnic backgrounds; this may have influenced the variability of the findings. Second, most studies assessed prenatal androgen exposure indirectly through the 2D:4D ratio, without direct hormonal or genetic measurements. To establish these relationships more definitively, further research involving larger and more diverse populations using standardized methods is needed.

## Author Contributions

**Tuba Güner Emül:** writing – review and editing, writing – original draft, supervision, methodology, investigation, data curation, conceptualization. **Emine Kaplan Serin:** writing – review and editing, writing – original draft, methodology, investigation, conceptualization.

## Funding

The authors have nothing to report.

## Ethics Statement

All procedures performed in studies involving human participants followed the institutional and/or national research committee's ethical standards and the 1964 Helsinki declaration and its later amendments or comparable ethical standards.

## Conflicts of Interest

The authors declare no conflicts of interest.

## Data Availability

The data that support the findings of this study are available from the corresponding author upon reasonable request.

## References

[ajhb70288-bib-0002] Barker, T. H. , J. C. Stone , K. Sears , et al. 2023. “The Revised JBI Critical Appraisal Tool for the Assessment of Risk of Bias for Randomized Controlled Trials.” JBI Evidence Synthesis 21, no. 3: 494–506. 10.11124/JBIES-22-00430. 36727247

[ajhb70288-bib-0003] Can, E. N. , and P. F. Türker . 2023. “Polycystic Ovary Syndrome.” Journal of the Faculty of Health Sciences, Başkent University 8, no. 3: 245–257.

[ajhb70288-bib-0004] Çatak, M. , F. Özsoy , and B. Demir . 2024. “Relationship Between the Likelihood of Suicide and Aggression Levels With 2D/4D Ratios in Patients Diagnosed With Polycystic Ovary Syndrome.” Journal of Experimental and Clinical Medicine 41, no. 4: 701–706.

[ajhb70288-bib-0005] Cattrall, F. R. , J. V. Beverley , and W. C. Gareth . 2005. “Anatomical Evidence for In Utero Androgen Exposure in Women With Polycystic Ovary Syndrome.” Fertility and Sterility 84, no. 6: 1689–1692. 10.1016/j.fertnstert.2005.05.061. 16359966

[ajhb70288-bib-0006] Centre for Reviews and Dissemination . 2008. Systematic Reviews: CRD's Guidance for Undertaking Reviews in Health Care. University of York ISBN 978‐1‐900640‐47‐3.

[ajhb70288-bib-0007] Deepika, V. , and P. Preethy . 2021. “Evaluation of Body Fat Composition and Digit Ratio (2D: 4D) in Polycystic Ovary Syndrome in Adolescents.” Current Health Sciences Journal 47, no. 3: 433–437. 35003777 10.12865/CHSJ.47.03.15 PMC8679143

[ajhb70288-bib-0008] Eltaweel, A. A. , A. M. Hamed , E. E. Sebaey , and D. M. Noor . 2018. “Second to Fourth Digit Ratio in Patients With Hirsutism and Its Correlation With Hormonal Assay.” Benha Journal of Applied Sciences 3, no. 1: 57–63.

[ajhb70288-bib-0009] Evin, F. , and İ. Ayrancı . 2024. “Evaluation of Digit Ratios in Youth With Polycystic Ovary Syndrome.” Cureus 16, no. 8: e67168. 39295659 10.7759/cureus.67168 PMC11408964

[ajhb70288-bib-0010] Haddaway, N. R. , M. J. Page , C. C. Pritchard , and L. A. McGuinness . 2022. “PRISMA 2020: An R Package and Shiny App for Producing PRISMA 2020‐Compliant Flow Diagrams, With Interactivity for Optimised Digital Transparency and Open Synthesis.” Campbell Systematic Reviews 18: 1230. 10.1002/cl2.1230. PMC8958186 36911350

[ajhb70288-bib-0011] Holden, R. R. , and G. C. Fekken . 2020. “Basic Personality Inventory.” In “Basic Personality Inventory”. Encyclopedia of Personality and Individual Differences, edited by V. Zeigler‐Hill and T. K. Shackelford . Springer.

[ajhb70288-bib-0012] Hönekopp, J. , and S. Watson . 2010. “Meta‐Analysis of Digit Ratio 2D:4D Shows Greater Sex Difference in the Right Hand.” American Journal of Human Biology 22: 619–630. 10.1002/ajhb.21054. 20737609

[ajhb70288-bib-0013] Karakoç, İ. 2024. “Polycystic Ovary Syndrome (PCOS) Pathogenesis, Diagnosis, and Common Treatment Options: A Literature Review.” Turkish Medical Student Journal 11, no. 1: 9–12.

[ajhb70288-bib-0014] Lujan, M. E. , A. J. Podolski , D. R. Chizen , D. C. Lehotay , and R. A. Pierson . 2010. “Digit Ratios by Computer‐Assisted Analysis Confirm Lack of Anatomical Evidence of Prenatal Androgen Exposure in Clinical Phenotypes of Polycystic Ovary Syndrome.” Reproductive Biology and Endocrinology 8, no. 1: 156. 21189149 10.1186/1477-7827-8-156 PMC3022844

[ajhb70288-bib-0015] Lujan, M. E. , G. B. Terri , R. C. Donna , C. L. Denis , and A. P. Roger . 2010. “Digit Ratios Do Not Serve as Anatomical Evidence of Prenatal Androgen Exposure in Clinical Phenotypes of Polycystic Ovary Syndrome.” Human Reproduction 25, no. 1: 204–211. 10.1093/humrep/dep363. 19855107 PMC2894079

[ajhb70288-bib-0016] Manning, J. T. , and B. Fink . 2023. “Digit Ratio (2D: 4D) and Its Relationship to Foetal and Maternal Sex Steroids: A Mini‐Review.” Early Human Development 183: 105799. 10.1016/j.earlhumdev.2023.105799. 37300988

[ajhb70288-bib-0017] Pandit, V. K. , M. Setiya , S. Yadav , and M. Jehan . 2016. “Digit Ratio (2D: 4D): A Potential Anatomical Biomarker for Predicting the Risk of Development of Polycystic Ovarian Syndrome.” IOSR Journal of Dental and Medical Sciences 15, no. 8: 58–64.

[ajhb70288-bib-0019] Roy, R. , R. Kundu , M. Sengupta , and P. Som . 2018. “Association Between Digit Length Ratio (2D: 4D) and Polycystic Ovarian Syndrome (PCOS)—A Study Among Eastern Indian Population.” Journal of the Anatomical Society of India 67: 14–19.

[ajhb70288-bib-0020] Teede, H. J. , C. T. Tay , J. J. E. Laven , et al. 2023. “Recommendations From the 2023 International Evidence‐Based Guideline for the Assessment and Management of Polycystic Ovary Syndrome.” Journal of Clinical Endocrinology and Metabolism 108, no. 10: 2447–2469. 10.1210/clinem/dgad463. 37580314 PMC10505534

[ajhb70288-bib-0021] Witchel Selma, F. A. , A. C. Burghard , R. H. B. Tao , and E. B. S. Oberfield . 2019. “The Diagnosis and Treatment of PCOS in Adolescents: An Update.” Current Opinion in Pediatrics 31, no. 4: 562–569. 10.1097/MOP.0000000000000778. 31299022

[ajhb70288-bib-0022] Witchel Selma, F. A. , E. O. Sharon , and S. P. Alexia . 2019. “Polycystic Ovary Syndrome: Pathophysiology, Presentation, and Treatment With Emphasis on Adolescent Girls.” Journal of the Endocrine Society 8, no. 3: 1545–1573. 10.1210/js.2019-00078. PMC6676075 31384717

[ajhb70288-bib-0023] Yan, X. , A. Zhu , Y. Li , et al. 2023. “Systematical Assessment of Digit Ratio in a Female Masculinization Disease: Polycystic Ovary Syndrome.” Frontiers in Endocrinology 14: 1146124. 37223048 10.3389/fendo.2023.1146124 PMC10202172

[ajhb70288-bib-0025] Yıldız, P. , M. Yıldız , A. C. Yıldırım , et al. 2015. “The 2nd to 4th Digit Length Difference and Ratio as Predictors of Hyperandrogenism and Metabolic Syndrome in Females.” Konuralp Medical Journal 7, no. 1: 45–49.

[ajhb70288-bib-0026] Zeng, X. , Y. J. Xie , Y. T. Liu , S. L. Long , and Z. C. Mo . 2020. “Polycystic Ovarian Syndrome: Correlation Between Hyperandrogenism, Insulin Resistance and Obesity.” Clinica Chimica Acta 502: 214–221. 10.1016/J.Cca.2019.11.003. 31733195

[ajhb70288-bib-0027] Zheng, Z. , and M. J. Cohn . 2011. “Developmental Basis of Sexually Dimorphic Digit Ratios.” Proceedings of the National Academy of Sciences of the United States of America 108, no. 39: 16289–16294. 10.1073/pnas.1108312108. 21896736 PMC3182741

